# P-196. Differences in *Clostridioides difficile* Molecular Epidemiology and Antimicrobial Susceptibility at Two Veterans Affairs Hospitals

**DOI:** 10.1093/ofid/ofae631.400

**Published:** 2025-01-29

**Authors:** Andrew M Skinner, Adam K Cheknis, Laurica A Petrella, Larry K Kociolek, Curtis Donskey, Jennifer Cadnum, Matthew H Samore, Dale N Gerding, Stuart Johnson, Charlesnika T Evans

**Affiliations:** University of Utah, Salt Lake City, Utah; Edward Hines Jr. VA Hospital, Hines, Illinois; Edward Hines Jr VA Hospital, Hines, Illinois; Ann & Robert H. Lurie Children's Hospital of Chicago, Chicago, IL; Cleveland VA Hospital, Cleveland, Ohio; Northeast Ohio VA Medical Center, Cleveland, Ohio; University of Utah, Salt Lake City, Utah; Edward Hines, Jr. Veterans Affairs Hospital, Hines, Illinois; Hines VA Hospital and Loyola University Medical Center, Hines, Illinois; Northwestern University and VA, Hines, Illinois

## Abstract

**Background:**

*Clostridioides difficile* infections (CDI) are caused by a diverse group of strains with differences in prevalence and differing antimicrobial susceptibilities. Over the past 20 years the *C. difficile* (CD) molecular epidemiology has changed as the prevalence of the epidemic strain recognized as PCR-Ribotype group (RT) 027 has decreased. The objective of this study was to determine the current molecular epidemiology and antimicrobial susceptibility patterns of CD at two Veteran Affairs hospitals.

**Figure 1.**
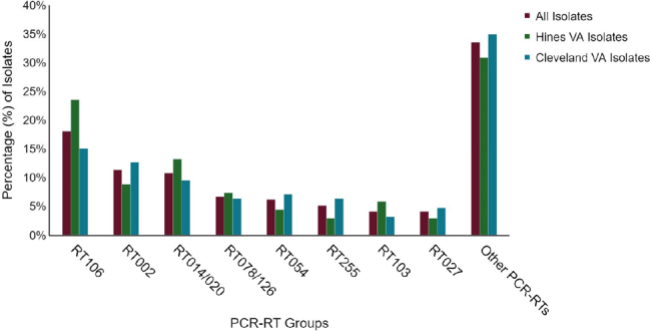
PCR-Ribotyping of Clostridioides difficile isolates collected from the Hines VA and Cleveland VA from 7/2022 - 6/2023

**Methods:**

We determined the molecular epidemiology and antimicrobial susceptibility of CD at the Edwards Hines Jr., VA hospital and the Louis Stokes Cleveland VA hospital from 7/2022 – 6/2023 from clinically relevant stool specimens. Available stools were cultured and recovered CD isolates underwent PCR-RT (n=194). *In vitro* minimum inhibitory concentration (MIC) was determined by agar gel dilution for 9 antibiotics known to precipitate CDI. Geometric mean MIC was compared between hospitals by Wilcoxon rank sum test and Kruskal-Wallis was used to compare MICs across PCR-RT groups.

**Figure:**
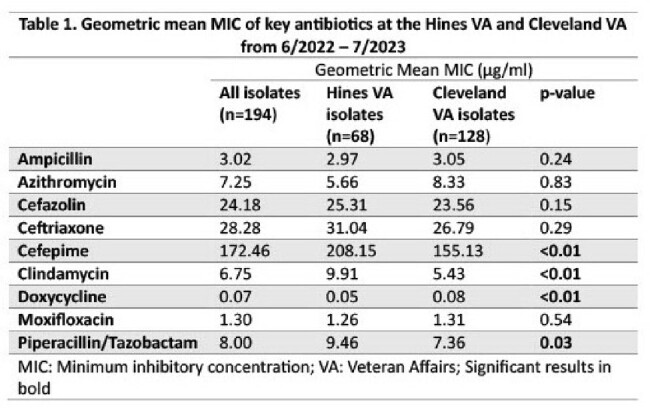

**Results:**

From 7/2022 – 6/2023, RT106 was the most prevalent strain accounting for 23.5% (16/68) and 15.1% (19/128) of isolates at the Hines VA and Cleveland VA, respectively. (Figure 1) RT002 and RT014/020 were the next most common strains, while RT027 accounted for only 4.1% (8/194) of all isolates. The Hines VA isolates had a higher geometric mean MIC for cefepime (208.15 µg/ml vs 155.13 µg/ml, p< 0.01), clindamycin (9.91 µg/ml vs 5.3 µg/ml, p< 0.01), and piperacillin/tazobactam (9.46 µg/ml vs 7.36 µg/ml, p=0.03). The Cleveland isolates had a higher doxycycline geometric mean MIC (0.08 µg/ml vs 0.05 µg/ml, p< 0.01). (Table 1) The clindamycin geometric mean MIC was elevated for RT255 (9.33 µg/ml) and RT027 (49.35 µg/ml) when compared to other groups (p< 0.01). The doxycycline geometric mean MIC was elevated for RT078/126 (1.17 µg/ml) when compared to other groups (p< 0.01). (Table 2)

**Figure:**
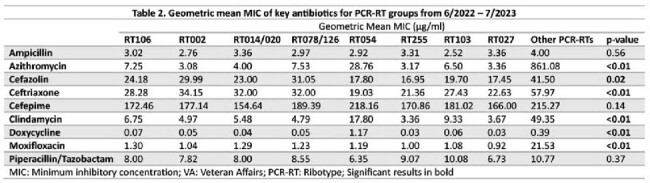

**Conclusion:**

The once epidemic strain RT027 has been supplanted by three different strain groups at both VA hospitals. Further research is needed to correlate local antibiotic usage patterns with the differing strain prevalence and antimicrobial susceptibilities noted.

**Disclosures:**

**Andrew M. Skinner, MD**, BioK plus: Advisor/Consultant|Ferring Pharmaceuticals: Advisor/Consultant|Recursion pharmaceutical: Advisor/Consultant **Larry K. Kociolek, MD, MSCI**, Merck: Grant/Research Support **Curtis Donskey, MD**, Clorox: Grant/Research Support|Pfizer: Grant/Research Support **Dale N. Gerding, MD**, AstraZeneca: Advisor/Consultant|Destiny Pharma: Advisor/Consultant|Destiny Pharma: Licensed IP to Destiny|Sebela: Advisor/Consultant|Sebela: Licensed IP **Stuart Johnson, M.D.**, Acurx Pharmaceuticals: Advisor/Consultant|Bio-K Plus International: Advisor/Consultant|Ferring Phamraceuticals: Advisor/Consultant

